# Movement control tests of the low back; evaluation of the difference between patients with low back pain and healthy controls

**DOI:** 10.1186/1471-2474-9-170

**Published:** 2008-12-24

**Authors:** Hannu Luomajoki, Jan Kool, Eling D de Bruin, Olavi Airaksinen

**Affiliations:** 1 Physiotherapie Reinach, 5734 Reinach, Switzerland; 2 University of Kuopio, Kuopio, Finland; 3 Institute of Physiotherapy, Department of Health, Zürich University of Applied Sciences, Winterthur, Switzerland; 4 Institute of Human Movement Sciences and Sport, ETH Zurich, Switzerland; 5 Department of Physical and Rehabilitation Medicine, University Hospital of Kuopio, Finland

## Abstract

**Background:**

To determine whether there is a difference between patients with low back pain and healthy controls in a test battery score for movement control of the lumbar spine.

**Methods:**

This was a case control study, carried out in five outpatient physiotherapy practices in the German-speaking part of Switzerland. Twelve physiotherapists tested the ability of 210 subjects (108 patients with non-specific low back pain and 102 control subjects without back pain) to control their movements in the lumbar spine using a set of six tests. We observed the number of positive tests out of six (mean, standard deviation and 95% confidence interval of the mean). The significance of the differences between the groups was calculated with Mann-Whitney U test and *p *was set on <0.05. The effect size (d) between the groups was calculated and d>0.8 was considered a large difference.

**Results:**

On average, patients with low back pain had 2.21(95%CI 1.94–2.48) positive tests and the healthy controls 0.75 (95%CI 0.55–0.95). The effect size was d = 1.18 (p < 0.001). There was a significant difference between acute and chronic (p < 0.01), as well as between subacute and chronic patient groups (p < 0.03), but not between acute and subacute patient groups (p > 0.7).

**Conclusion:**

This is the first study demonstrating a significant difference between patients with low back pain and subjects without back pain regarding their ability to actively control the movements of the low back. The effect size between patients with low back pain and healthy controls in movement control is large.

## Background

Movement impairment syndromes are important for physiotherapists when we consider that the detection of faulty movement or kinesiopathology is a key competence of physiotherapy [1]. In the past, kinesiopathologic movement patterns in the lumbar spine have been investigated and described [1-5], resulting in the publication of both reliability and validation studies of the examination procedures used [6-12]. However, there is limited evidence of a difference between movement patterns in patients with low back pain (LBP) and individuals without LBP.

The underlying hypothesis is that impaired movement control (MC) and a lack of awareness of maladaptive movement patterns perpetuates LBP. Physiotherapists make clinical decisions based on the observation of movement control. O'Sullivan [4] describes back pain patients with reduced MC and excessive movement as pain provocateurs. Sahrmann [1] suggests in her theory of "relative flexibility" that movement occurs through the pathway of least resistance, e.g. if hip motion is relatively stiff compared to that of the low back, then movement is more likely to occur in the back, leading to a back pain problem related to the direction of that particular movement. Synonyms used for movement impairment syndromes are motor control dysfunctions [2,3] and MC impairment [4,13].

Reliable observation of variations in the movement control of the low back in patients with LBP is important [1,2,4]. In a Delphi study of American physical therapists who were Orthopaedic Clinical Specialists or Fellows of the American Academy of Orthopaedic Manual Physical Therapists (N = 168) [14], 88% of the specialized therapists rated abnormal movement patterns as the main finding in clinical instability of the low back. Maladaptive movement control can also occur with hypomobility. To our knowledge, however, no study has examined whether there is a difference in movement control ability between patients with LBP and healthy controls.

The reliability of movement control tests has been evaluated in earlier studies. Dankaerts et al. [15] reported an almost perfect agreement (k = 0.96 and percentage agreement 97%) between two expert examiners rating a motor control dysfunction classification. Van Dillen et al. [9] used a whole package of physical examination items in order to categorize the patients in an impairment dysfunction subgroup. They found a very high agreement for the assessment of symptoms among the examiners (k > 0.89 and percentage agreement > 98%). Furthermore, they examined the reliability of observation of spinal alignment and movement. In general the interpretation of the spinal alignment was slightly lower (k = 0.27–0.58) than for the observation of active movements (k = 0.26–1.00). Luomajoki et al. [16] examined ten movement control tests for the back. Four blinded physiotherapists evaluated subjects through observation of videos. For the intra-observer reliability, five tests out of ten showed an excellent reliability (k > 0.80). Four further tests had a substantial reliability (k = 0.6–0.8) and one was moderate (0.51). Five out of ten tests showed a substantial inter-observer reliability (k > 0.6), four tests had Kappa values between 0.4 and 0.6 (good) and one test was under 0.4 (fair). The percentage agreement varied between 65% – 97.5%. White & Thomas [12] investigated the reliability (N = 37) of 16 tests of the Movement System Balance approach developed by Sahrmann, finding a satisfactory reliability between raters. However, the difference between movement patterns in patients with LBP and individuals without LBP received little attention from these previous studies. Murphy et al. [17] (N = 42) investigated one test, namely prone hip extension, that was rated positive if the lower back moved when the hip was extended. Inter-rater reliability was substantial with k = 0.72 for left and 0.76 for right hip. This test is different when compared with the prone knee bend test in that it examines active extension control of the lower back. Table 1 gives an overview of the reliability studies published before.

**Table 1:** The intertester reliability of the movement control tests of the low back (Kappa coefficient values)

Test	Luomajoki et al 2007	Van Dillen et al 1998	White & Thomas 2002
Waiters bow (flexion control)	0.62		

Pelvic tilt (extension control)	0.65		

One leg stance (rotation/lateral flexion control)	0.54		

Sitting knee extension (flexion control)	0.72	0.58	0.17

Rocking 4 point kneeling (flexion control)	0.57	0.78	0.62

Rocking 4 point kneeling (extension control)	0.68	0.51	0.39

Prone knee bend (extension control)	0.47	0.76	0.22

Prone knee bend (rotations control)	0.58	0.43	

According to Sackett [18], phase 1 of diagnostic research compares test results in patients and control individuals. Ideally, healthy persons should test negative and affected persons test positive. Because LBP is a multidimensional problem, not all patients need to have problems with MC. On the other hand, if both healthy controls and patients with LBP have impaired movement control, the clinical importance of impaired MC is limited and research on diagnosing MC would not be worthwhile. According to Sackett [18] this first phase of evaluation of a diagnostic test "*can not be translated into diagnostic action but adds to our biological insight into mechanisms of disease and may serve later research into treatment as well as diagnosis*". By the nature of clinical instability, there is so far no gold standard for movement control of the low back. In order to measure the concurrent validity, a test should be available which can be compared to the actual test used. This situation is frequent as gold standards are not available for many diagnostic clinical tests. Previous examples of phase 1 testing of clinical tests were related to the diagnosis of the patellofemoral syndrome [19,20] and shoulder impingement [21]. For both conditions, as in impaired lumbar MC, no gold standard is available.

As the reliability of the movement control test battery of the low back in our earlier study was shown to be acceptable to substantial for 6 tests, the next step is to evaluate whether there is a difference in movement control between patients with low back pain and healthy controls in this 6 tests battery. The aim of this study was to determine whether the number of positive tests out of six active MC tests was different in patients with a wide time range (acute, sub-acute and chronic) of diagnosed LBP compared with healthy controls and to determine the effect size of the differences. Furthermore, we wanted to explore whether there were differences in the numbers of positive tests depending on the duration of LBP.

## Methods

### Study design

**Figure 1:**
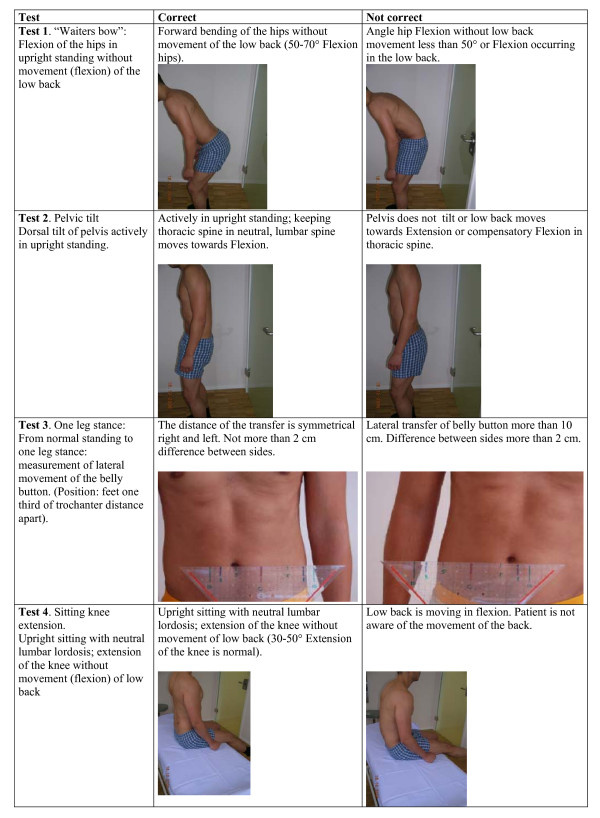
**Test set description; tests 1. – 4**.

**Figure 2:**
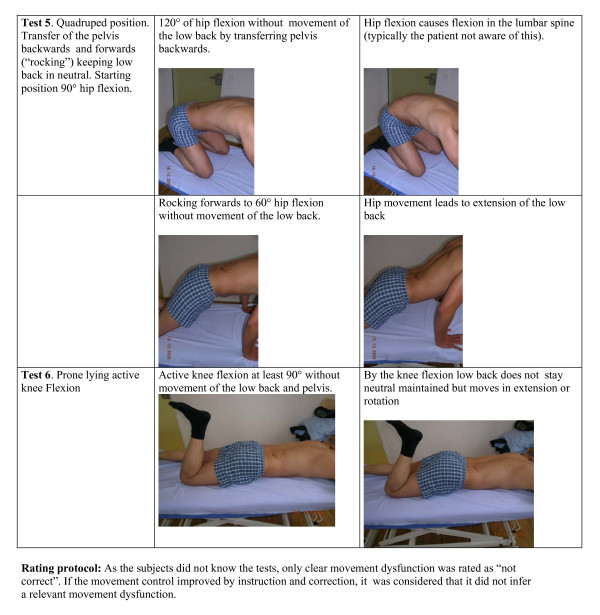
**Test set description; tests 5. – 6**.

This was a case control study applying six active MC tests for the lower back in patients with LBP and healthy controls. As the MC tests are direction specific, a battery of tests is required for a comprehensive clinical assessment. We created a test battery of six tests (Figures 1, 2) for which the reliability has been shown to be at least acceptable (Table 1). Subjects performed the set of tests in a standardized manner. 12 physiotherapists participated in rating the tests' results of the patients as either positive or negative. The research was approved by the ethics committee of the government health authorities of Canton Aargau, Switzerland, and written informed consent was obtained from all patients.

The sample size was calculated for continuous outcome variables. Choosing the level of significance as alpha = 0.05 and power (beta = 0.80) for testing Ho: Group1 = Group2 versus H1: Group1≠Group2, the required sample size for group testing would be 99 cases per group for an effect size of d>0.5 [22]. The sample size was set as N = 105 subjects in each group to cater for a potential dropout rate of 5%.

### Setting

Subjects were examined in five outpatient physiotherapy clinics in Switzerland (Canton Aargau) between July 2006 and May 2007.

### Subjects

210 subjects, 108 patients with non-specific LBP and 102 control subjects without back pain were included in the study. Selection of consecutive patients was carried out by participating physiotherapists. Inclusion criteria for patients were non-specific low back pain (NSLBP), and to have been referred to physiotherapy by a physician due to the back pain. NSLBP has been described by Waddell [23] as "simple back pain", which has a mechanical nature; the pain is situated in lumbosacral region, buttocks and thighs. Exclusion criteria were serious pathologies such as unhealed fractures, tumours, acute trauma, serious illnesses or positive neurological findings. The patients also had to be able to understand the instructions in German. Healthy controls were volunteers who did not have any back pain at that time or three months prior to the testing and were comparable in age and gender. These subjects were friends, colleagues or family members of the participating physiotherapists, they were currently not in a medical or physiotherapy treatment, but some did have some musculoskeletal problems when asked about their health status (Table 2.).

**Table 2:** Background characteristics of the subjects

	Patients with LBP N = 108	Healthy controls N = 102	Sig.
Age years (Mean, SD)	41 (15)	37 (12)	0.08*

Height cm (Mean SD)	169 (9)	171 (9)	0.65'

Weight kg (Mean SD)	67 (11)	67 (12)	0.21'

Male (N, %)	36 (33%)	44 (43%)	0.81"

Female (N, %)	72 (67%)	58 (57%)	0.81"

Working	71 (65%)	51 (50%)	0.16"

Retired	15 (14%)	15 (15%)	0.86"

Student	12 (12%)	33 (27%)	0.02"

Disability allowance	10 (9%)	0	<0.01"

Sport (>2/week)	45 (42%)	52 (51%)	0.58"

Other musculoskeletal problems all	38 (34%)	37 (36%)	0.87"

* Mann-Whitney U test for non-parametric distribution.

' t-test for parametric distribution.

" Chi square test for nominal data.

### Raters

12 physiotherapists examined the subjects. The physiotherapists had on average seven years (SD = 2.3) of working experience and participated in a two-and-a-half year postgraduate manual therapy specialization program including a three day course for the assessment and treatment of MC dysfunctions. Raters were trained in the test procedure using instruction, patient cases and rating of videotaped tests. Criteria were discussed and typical dysfunctions were presented. Physiotherapists were not blinded to the subjects' group.

### Test procedure

Physiotherapists scored the performance of the subjects on the six MC tests resulting in a score of 0–6 positive tests (Figure 1). Subjects had never performed the tests before and received standardized instructions, for example in the prone knee bend test the instructions were: "Please bend your knee as far as you can without moving your back", and: "keep your back in the same position, do not let it move while bending the leg". If the patient did not understand how to perform the test, it was explained again and demonstrated by the examiner. Three trials were permitted. The order of the tests was always the same (standing, sitting, quadruped, prone), in order to ensure that all subjects were assessed the same way and under the assumption that this procedure would mimic clinical reality. Patients wore only underwear to allow the observation of the entire spine, hips and lower extremities.

### Statistical analysis

Data was analyzed with SPSS 14.0 for Windows. The comparability of the groups was tested with unpaired t-tests (Table 2) for parametric variables and the equivalent non parametric test where appropriate. The Mann-Whitney U test was used for ordinal and the chi square test for nominal variables. We compared the mean number of positive tests in the two groups. The differences between the groups were analyzed by the effect size (ES) d. The ES (d) is the difference of the means divided by the mean standard deviation of the groups. ES with d<0.2 are considered small, d>0.5 moderate and d> 0.8 large [23]. The significance of the differences between the groups was calculated with an unpaired Mann-Whitney U test and p was set on <0.05. We also performed a subgroup analysis of the number of positive tests depending on LBP duration with the Kruskal Wallis test. The Mann Whitney U test was used to test for differences between the groups using Bonferroni correction (alpha = 0.016).

## Results

108 patients with NSLBP and 102 controls without LBP were included in the study. Tables 2 and 3 show the descriptive data of the subjects. The groups were comparable in age, gender, height and weight (Table 2.). In their sociodemographic background there was a difference in working status (healthy controls having less time off work). The control group had more students than the LBP group and no one received a disability allowance. Participants in both groups had other musculoskeletal problems which were assessed by interview (e.g. "Do you have any other problems apart from your back?" "Yes, my elbow hurts when I play tennis"). A comparable number of subjects in both groups were participating in sports. All subjects completed the examination according to protocol.

**Table 3:** Characteristics of LBP patients

All	N = 108 (100%)
Acute LBP (< 6 weeks)	29 (27%)

Subacute LBP (6–12 weeks)	30 (28%)

Chronic LBP (>12 weeks)	46 (45%)

Local back pain without leg pain	51 (47%)

Leg pain	62 (57%)

Mean Score Roland Morris Questionnaire (max 24, mean, % and SD)	8 (33%, 5)

**Figure 3:**
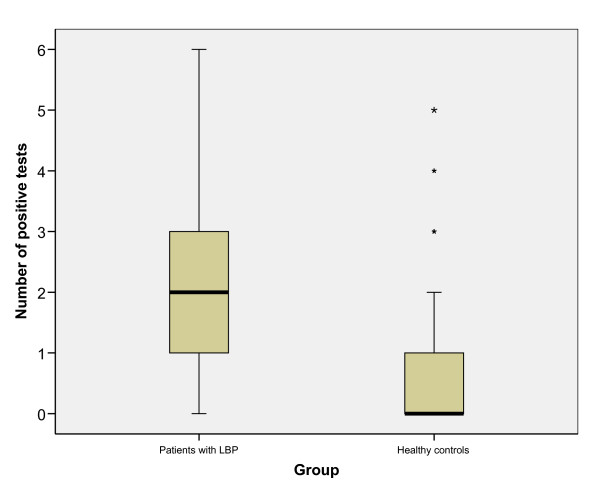
**Number of positive tests in the two groups**.

There were no adverse effects of test performance. On average, the number of positive tests out of six was 2.21 (95%CI: 1.94–2.48) in patients with LBP and 0.75 (95%CI: 0.55–0.95) in healthy controls. The ES (d) for the difference between the groups was 1.18 (95%CI: 1.02–1.34) (Table 4). The statistical test showed that this was a significant difference (p < 0.001). Figure 3 shows the difference between the groups.

**Figure 4:**
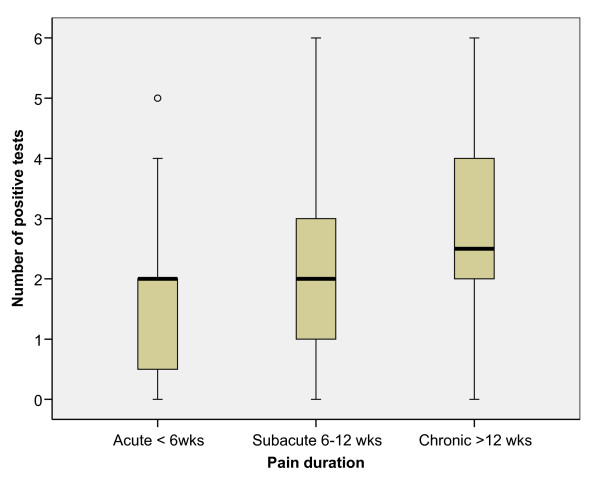
**Number of positive tests depending of the duration of LBP**. The difference between acute and chronic (p < 0.01) and between subacute and chronic (p < 0.03) was significant but not between acute and subacute (p > 0.7) patient groups.

We performed a subgroup analysis of the number of positive tests depending on pain duration (Figure 4). A Kruskal Wallis test showed a significant difference between the groups (p < 0.02). According to the Mann Whitney U test, there was a significant difference between acute and chronic (p < 0.01), as well as between subacute and chronic (p < 0.03) but not between acute and subacute (p > 0.7) patient groups.

## Discussion

This is the first study demonstrating a clear difference between patients with LBP and subjects without back pain regarding their ability to actively control the movements of the low back. There is also a significant difference depending on pain duration. Patients with chronic LBP have significantly more positive tests than those with acute or subacute LBP.

We used a test battery of six tests for which acceptable reliability has been demonstrated in our previous research [16], in which we evaluated ten movement control tests. We refrained from testing the six movements in a random order because we assume that this procedure best represents clinical practice where routines are often developed. This procedure has the advantage that the chance of behavioural responses being altered by differences in prior test history decreases. A limitation of this procedure, however, is that we are unable to define whether the order of testing influences patient performance on subsequent tests.

The face validity of the six direction specific tests in this study (see Figures 1 &2) is supported by the following considerations. The tests "waiters bow", "sitting knee extension" and "rocking on all fours backwards" assess flexion movement control. These tests, where hip flexion is expected while the lumbar spine is stabilized, are positive if flexion in the lumbar spine occurs. Similarly, extension movement control is assessed in the tests "pelvic tilt", "rocking all four forwards" and "prone knee bending" where the subject should extend the hip while the lumbar spine is stabilized. The "one leg stance" test is testing lateral flexion and rotation control. During lateral weight shift ab- and adduction in the hip joints should occur in the hips while the lumbar spine maintains neutral position.

Face validity also relates to the subject's acceptance of a test. Patients will sometimes resist taking a test if it does not appear to be related to something they can understand and accept e.g. performing movements of the back in relation to LBP complaints. Face validity can be important in winning a patient's cooperation in a testing situation [24]. The patient's acceptance of the six tests during our study was good. No volunteer in our study resisted taking a test because he/she felt that the test would "not make sense".

Two other studies have at least partly evaluated the reliability of the same tests [9,16] (Table 1.). The results by van Dillen et al. [9] and Luomajoki et al. [16] were similar whereas those by White & Thomas [12] reported lower reliability coefficients for these tests. These contradictory findings might be attributable to differences in test instruction procedures for the assessors. The van Dillen group, as one of the developers of this test, has previously been criticized because they were very carefully training their assessors. This intensive training might have biased the results. In Thomas & White's [12] study, one pair of assessors had a three day course by the test developer and another pair received only written information. They also used the tests as a provocation test, which might have lead to lower reliability because after the first test the subject anticipates that it will hurt and therefore moves differently in the second assessment. In our study the 12 participating assessors were students of a 2.5 years training program specializing in musculoskeletal/manual therapy and had taken a three day course on movement control issues. It would therefore seem that the amount of education in musculoskeletal physiotherapy provides a better intertester reliability in the test evaluation. These conflicting findings on interrater reliability, where the experience of the assessing physiotherapist seemingly plays an important role, should have clear clinical implications. If more than one therapist in a clinical setting is going to record data on a patient, then it is important that all therapists concerned apply the tests consistently and reliably. If this cannot be guaranteed then the data is of little use. Clinicians specialized in musculoskeletal physiotherapy and with comparable levels of practical experience that are evaluating movement control dysfunctions in the same patients with LBP can, however, use these six tests in their everyday practice with confidence.

The difference between the groups was significant (p < 0.001). On average, patients with LBP had 2.21 (95%CI: 1.94–2.48) positive tests against 0.75 (95%CI: 0.55–0.95) for healthy controls. The ES between the groups was large; 1.18 (95%CI: 1.02–1.34), meaning that there is a large difference in movement control between subjects with and without back pain.

Our subgroup analysis revealed that there are differences between the subgroups in relation to the duration of the LBP. There was a significant difference between acute and chronic (p < 0.01) as well as between subacute and chronic (p < 0.03) but not between acute and subacute (p > 0.7) patient groups. It appears that the longer the symptoms of LBP last, the worse the movement control becomes.

These findings deliver some indication of the construct validity of the six tests that we used. Construct validity in this study relates to the hypothetical construct of impaired MC. It is assumed that MC can be inferred from movement behaviour exhibited with the six tests. Our evaluation shows that the summed value of the six tests has the potential to discriminate between patients with LBP and healthy controls. This is a first indication that the battery of six tests might have the adequate construct validity necessary for use as a clinical instrument. Future research on the classification accuracy of these tests is, however, necessary to substantiate this assumption.

Some other limitations of the study must also be mentioned. As so far no gold standard has been defined for MC of the low back, it is impossible to determine the sensitivity and specificity of the test battery that we used. The underlying hypothesis of the MC tests is that the low back is not moving during the test. A gold standard for checking this assumption would be by using functional x-rays, functional MRI or electronic movement measuring devices. Our focus was only to examine whether the subjects could control the neutral position of the back during the tests. The assessor's decision was based solely on their own observation, which was subjective. Future research should also use more objective measurement tools to see whether the lumbar spine really stayed neutral during the tests. A comparable gold standard might be functional radiography or movement analysis systems such as Vicon^® ^or Optotrac^®^. Future research could also address whether there is a difference between the range of motion in negative and positive tests to see if these patients also have a hypermobility as it could be hypothesized by a clinical instability.

Another limitation of our study is that examiners were not blinded to the subjects' group. This might have introduced a major bias in the results as the clinicians may have been influenced in their judgments by their expectations. However, blinding is very difficult because in spite of blinding clinicians are likely to identify patients based on the observation of pain related behaviour.

The only difference in the selection criteria between the groups was whether subjects had low back pain or not [18]. This study demonstrated that there is a difference between subjects with and without back pain which is a first step in the validation process of developing diagnostic tests. In clinical practice identifiable subgroups of patients with LBP have been proposed [3,13,14], e.g. flexion, extension, rotational pattern or combinations of them, that are distinguishable from one another based on MC problems. Future studies should investigate whether the six tests evaluated in this study are able to distinguish these subgroups. The correlation between MC ability and other findings, such as disability and pain, should be evaluated in future studies. Furthermore, research is needed to look into whether or not improvement of MC ability is causally related to symptom reduction.

One might state that the observed differences between the group of LBP and non-LBP individuals are the result of prior experiences. LBP patients will most probably have been examined for their back complaints many times during the course of their disorder, whereas the non-LBP individuals are likely to be new to the MC testing. This fact could also explain the observed differences. However, if this were the case it would seem logical that those with most experience in performing test movements, i.e. the LBP patients, would also perform the test better due to previous learning than the naïve individuals. This, however, clearly was not the case in our study.

There are possible confounding factors influencing the performance of the tests. In spite of standardization, the instructions of the physiotherapists as well as the observation and interpretation of performance may have been slightly different among assessors. In addition to impaired movement control, neuromechanosensitivity (test 4) or muscle length (test 6) may have influenced test performance.

LBP is a multidimensional phenomenon and, consequently, MC alone cannot be expected to explain back pain. However, in this first stage of validation of a diagnostic test battery we demonstrated that a group of six clinically applicable tests shows a clear difference between groups of patients with LBP and non-LBP controls. Only five out of 102 healthy persons had three or more positive tests. This could be explained by the fact that not everyone has good movement coordination ability – like not everyone can dance.

To our knowledge, no other study has compared the MC test battery in patients with LBP and healthy controls. Several studies have, however, been published on other aspects of motor control [25-31], with movement control being one subcategory of motor control. Muscle diameter, recruitment patterns of individual muscles, movement tests and volitional movement all measure different aspects of motor function. Electromyography and kinematic assessment may be of additional value for the assessment of motor control in physiotherapy practice settings.

Van Dillen et al. [11] performed a cross sectional, construct validity study on mechanical LBP of 188 patients. They were interested in finding categories of movement system impairment based syndromes. A history was taken and a subsequent physical assessment that included 28 different movement items was performed. Approximately 50% of the variance in the patients' responses to the impairment tests could be explained by three factors: lumbar extension with rotation, extension and lumbar rotation syndromes as described by Sahrmann [1]. Their study clarified how the direction of MC explained the back pain problem experienced by the patients. Our study, on the other hand, demonstrated that there is a difference between patients and healthy controls in MC.

While subgrouping of non specific LBP is an important issue [31], it is debatable whether a dysfunction in the movement control is a subgroup of LBP itself. It might also form a part of the diagnosis of clinical instability, a term which was first introduced by Panjabi [32,33]. The basic idea is that the spinal stability relies on three subsystems i.e. the passive system, the active system and the neural control system. This theory, where the neural control system controls the movements, has found wide acceptance and these different subsystems have already been studied to a certain extent. Cook [14] has established the clinical pattern of the clinical instability of the low back through a qualitative Delphi study. 168 in manual therapy or to musculoskeletal physiotherapy specialized therapists were asked about the diagnosis of clinical instability and the majority of the participants agreed to a great degree (88%) that the most important physical findings are poor co-ordination, proprioception and control of the active movements, which links it directly to this study. Currently, movement control tests are widely discussed [2-5] and many physiotherapists around the world are using movement control tests in their evaluation of patients with LBP.

Further studies are needed to establish the concurrent validity of the movement control tests. A comparison with a gold standard is needed. Is there a certain subgroup of LBP suffering from movement control dysfunction? Finally, outcome studies of patients with non specific low back pain and movement control dysfunctions are of great interest.

## Conclusion

This is the first study demonstrating a significant difference between patients with LBP and subjects without back pain regarding their ability to actively control the movements of the low back. The ES between patients with LBP and healthy controls in MC is large with 1.18 (95%CI: 1.02–1.34). There is also a significant difference in MC depending on pain duration. Patients with chronic LBP have significantly more positive tests than patients with acute or subacute LBP. This first phase of evaluation of a diagnostic test can not be translated into diagnostic action but adds to our biological insight into mechanisms of dysfunction and may serve later research into treatment as well as diagnosis.

## Competing interests

The authors declare that they have no competing interests.

## Consent

A written informed consent was obtained from the patient for publication of this case report and accompanying images. A copy of the written consent is available for review by the Editor in Chief of this journal.

## Authors' contributions

HL accumulated the data, calculated statistics, and was the main writer of the paper. JK was involved in the planning of the study, methodological considerations, analysis of the data, and critically revised the manuscript for its content. EdB was involved in the planning of the study, methodological considerations, and critically revised the manuscript for its content. OA was involved in the planning of the study, methodological considerations, and revised the paper. All authors read and approved the final manuscript.

## Pre-publication history

The pre-publication history for this paper can be accessed here:


